# RETRACTION: Comparing the Efficacy of Chlorhexidine and Povidone–Iodine in Preventing Surgical Site Infections: A Systematic Review and Meta‐Analysis

**DOI:** 10.1111/iwj.70428

**Published:** 2025-03-26

## Abstract

**RETRACTION:** BaiD. , ZhouF. , and WuL. , “Comparing the Efficacy of Chlorhexidine and Povidone–Iodine in Preventing Surgical Site Infections: A Systematic Review and Meta‐Analysis,” International Wound Journal 21, no. 2 (2024): e14463, 10.1111/iwj.14463.PMC1082852437885342

The above article, published online on 27 October 2023, in Wiley Online Library (http://onlinelibrary.wiley.com/), has been retracted by agreement between the journal Editor in Chief, Professor Keith Harding; and John Wiley & Sons Ltd. Following an investigation by the publisher, all parties have concluded that this article was accepted solely on the basis of a compromised peer review process. The editors have therefore decided to retract the article. The authors did not respond to our notice regarding the retraction.

**RETRACTION:** D. Bai , F. Zhou , and L. Wu , “Comparing the Efficacy of Chlorhexidine and Povidone–Iodine in Preventing Surgical Site Infections: A Systematic Review and Meta‐Analysis,” International Wound Journal 21, no. 2 (2024): e14463, 10.1111/iwj.14463.PMC1082852437885342

The above article, published online on 27 October 2023, in Wiley Online Library (http://onlinelibrary.wiley.com/), has been retracted by agreement between the journal Editor in Chief, Professor Keith Harding; and John Wiley & Sons Ltd. Following an investigation by the publisher, all parties have concluded that this article was accepted solely on the basis of a compromised peer review process. The editors have therefore decided to retract the article. The authors did not respond to our notice regarding the retraction.

